# Metallic mercury use by South African traditional health practitioners: perceptions and practices

**DOI:** 10.1186/s12940-015-0053-4

**Published:** 2015-08-15

**Authors:** Renée A. Street, Gaëtan M. Kabera, Catherine Connolly

**Affiliations:** HIV Prevention Research Unit, South African Medical Research Council, Durban, South Africa; Discipline of Occupational and Environmental Health, School of Nursing and Public Health, University of KwaZulu-Natal, Durban, South Africa; Biostatistics Unit, South African Medical Research Council, Durban, South Africa

**Keywords:** Mercury, Traditional medicine, Traditional health practitioners, Socio-cultural practices

## Abstract

**Background:**

Mercury is a toxic metal however its use in traditional healthcare systems remains widespread. The aim of this study was to determine the prevalence of mercury use by South African Traditional Health Practitioners (THP) and to document reasons for use and administration methods.

**Methods:**

A cross-sectional study design was employed. A total of 201 THPs were enrolled from two main metropolitan areas of KwaZulu-Natal (South Africa), and 198 were included in the final analysis. Information on demographic characteristics, reasons for using or not using mercury as well as mercury administration methods were collected.

**Results:**

Of the 198 THPs, 78 (39 %) used mercury for healing purposes and 74 (95 %) of the mercury users stated that they were taught to use it by another THP. The two main routes of administration were oral and sub-cutaneous implantations (ukugcaba) at 85 % (*n* = 66) and 59 % (*n* = 46), respectively. The most common responses for mercury administration were for child birth (*n* = 70; 90 %) and protection against guns (*n* = 39; 50 %).

**Conclusion:**

This is the first study to describe the prevalence and practice of mercury use in South African traditional medicine. Socio-cultural mercury use is a potential source of exposure to both THPs and their patients. In light of such findings, public education messages and regulatory measures need to be effected.

## Background

Mercury is a naturally occurring metal which exists in three main forms: elemental (or metallic), inorganic (e.g. mercuric chloride) and organic (e.g. methyl- and ethyl mercury) [1]. It is a toxic, tenacious pollutant and human exposure to mercury is caused mainly by occupational exposure, ingestion of contaminated fish or mercury outgassing from dental amalgam fillings [1]. Chemical form, dosage, exposure period and route as well as stage of individual human development are all aspects determining the incidence and severity of adverse human effects [2, 3].

The inimitable appearance and peerless properties of metallic mercury have attracted human attention throughout history [4]. Liquid at room temperature, metallic mercury evaporates rapidly and forms a colourless, odourless vapour [5] with the foremost exposure route of metallic mercury being inhalation of these mercury vapours [6]. Reports of subtle nervous system toxicity have been documented in workers exposed to a low elemental mercury level in the air (≥20 μg/m^3^) and renal changes have been observed at higher exposure levels [1, 3]. However dose–response relationships are not as well defined for other organs [1].

In efforts to eliminate mercury-related diseases, the World Health Organisation (WHO) has emphasized the need to identify traditional practices involving mercury [3]. Mercury for therapeutic purposes was prevalent until the 20th century when the detrimental effects of its exposure became notorious [7, 8]. Nonetheless, mercury in traditional healthcare systems is still widespread today [3, 5, 9]. For example, in traditional Chinese medicine, mercury is part of certain preparations under the colloquial names of ‘*cinnabaris*’ (mercuric sulfide) and ‘*calomel*’ (mercurous chloride) [10]. In certain Caribbean and Latin American traditions, mercury, known as ‘*azogue*’ , is used for a range of cultural and religious practices linked to healthcare including the use of mercury to treat intestinal disorders [11].

With a diverse range of cultural and socioeconomic backgrounds, approximately 25 million South Africans use traditional medicines [12]. South African traditional health practitioners (THPs) can be grouped according to healing type. For example, a diviner (*sangoma*) specialises in divination and acts as an ancestral spirit medium; a herbalist (*inyanga*) has extensive knowledge of medicinal plants and a faith healer (*umthandazi*) heals mostly with prayer and holy water. Each group has specific attributes however roles and responsibilities often overlap [13, 14]. South African traditional medicines are commonly plant-derived materials however the addition of inorganic substances as therapeutic agents have been documented [15]. To date there is no information regarding mercury use in South African traditional medicine. The aim of this paper is to determine the prevalence of mercury use by THPs of KwaZulu-Natal Province (South Africa) and among those using mercury for healing purposes, to determine reasons for therapeutic administration as well as mode of administration.

## Methods

This study is part of a larger study which aimed to survey commonly used inorganic substances in traditional medicine. Such substances are collectively known as *imikhando* in isiZulu; the literal translation of which is *‘ore’*. The results of mercury practice and perceptions by THPs responding to their current use of mercury for healing purposes are reported in this paper.

### Sampling

**Fig. 1:**
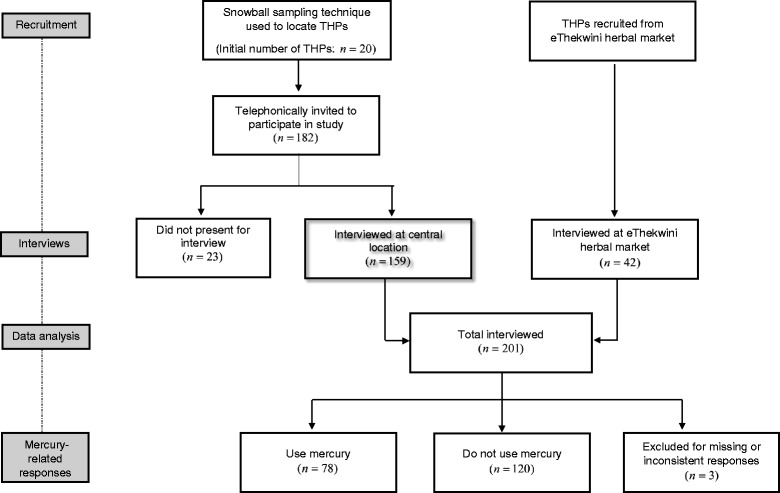
Flow diagram of study design

THPs were sourced from two main metropolitan areas of KwaZulu-Natal Province, South Africa; eThekwini (Durban) and uMgungundlovu (Pietermaritzburg). Because no official THP database exists, snowball sampling method was utilized with an initial total sample of 20. All THPs were in practice with active and recognized consultation sites; this information was verified by district THP coordinators assisting in the study. After potential participants were identified, telephonic contact was initiated to inform and invite participants to the study. Some participants who accepted the invitations did not present themselves to the study thus from the initial 182 invited, 159 (87 %) were interviewed at a centralized location. To increase heterogeneity, a further 42 THPs practicing at the eThekwini herbal market were invited to partake in the study and were interviewed at their place of consultation at the market. Hence 201 THPs were interviewed (Fig. 1). All interviews were conducted between April and May 2012. The participants signed an informed consent form prior to the start of the interview, and structured questionnaires were administered in the local language (isiZulu) by trained interviewers.

## Ethical approval

The study was approved by the Biomedical Research Ethics Committee (BREC) at the University of KwaZulu-Natal (BREC BF185/010).

### Statistical analysis

Data collected using a questionnaire presented to 201 THPs were captured on an Excel spreadsheet, then exported to STATA (version 13.1) for analysis. Two participants were excluded from analysis since they had missing information in all relevant questions and one more participant was excluded from further analysis because of inconsistent responses. Demographics, reasons for mercury use as well as modes of administration were analysed using statistics such as medians (inter-quartile ranges) for continuous variables and frequencies (percentages) for categorical variables. For certain questions, multiple responses were allowed. Wilcoxon rank sum test was used to compare medians of continuous variables. The degree of association between categorical variables was assessed using Pearson chi-square test or Fisher’s exact test, where appropriate.

## Results

Table 1 contains the demographic characteristics of the 198 respondents included in analysis, cross tabulated by mercury users and non-mercury users. The results in the first column (total) indicate that of 198 THPS, 168 (85 %) were from eThekwini and the remaining THPs were from uMgungundlovu. The majority of respondents were female (*n* = 141; 71 %). Most of the THPs had a single healing practice (*n* = 137; 69 %) whilst 60 (30 %) THPs were multidisciplinary. The main type of healing practice for the THPs was diviner (sangoma) (*n* = 101; 51 %). The median practice period was found to be 8 years (IQR 3–18), with 62 % of the THPs having experience of more than 5 years. The level of education ranged from none (16 %) to having attended tertiary education (3 %).

**Table 1:** Demographic characteristics of study participants

	Total N =198	Mercury users N =78	Non- mercury users N =120	
Characteristics	n (%)	n (%)	n (%)	*p*-value
Practice area				0.25^†^
Durban	168 (84.9)	69 (88.5)	99 (82.5)	
Pietermaritzburg	30 (15.1)	9 (11.5)	21 (17.5)	
Gender				0.26^†^
Female	141 (71.2)	52 (66.7)	89 (74.2)	
Male	57 (28.8)	26 (33.3)	31 (25.8)	
Type of healing practice^a^				0.24^¤^
Diviner (*Sangoma*)	101 (51.3)	36 (46.8)	65 (54.2)	
Herbalist (*Inyanga*)	27 (13.7)	14 (18.2)	13 (10.8)	
Diviner/faith healer	27 (13.7)	7 (9.1)	20 (16.7)	
Herbalist/diviner	19 (9.7)	10 (12.9)	9 (7.5)	
Herbalist/faith healer	8 (4.6)	4 (5.2)	4 (3.3)	
Faith healer (*Mthandazi*)	6 (3.1)	1 (1.3)	5 (4.2)	
Diviner/THP trainee	4 (2.0)	2 (2.6)	2 (1.67)	
THP trainee (*Thwasa*)	3 (1.5)	2 (2.6)	1 (0.8)	
Diviner/faith healer/THP trainee	1 (0.5)	0 (0.0)	1 (0.8)	
Herbalist/diviner/faith healer	1 (0.5)	1 (1.3)	0 (0.0)	
Years of practice^b^				
Median (IQR)^d^	8 (3–18)	9 (2–19)	8 (4–17)	0.88^°^
Years of practice in 5 year intervals^a^				0.21^†^
≤5 years	74 (38.0)	31 (40.3)	43 (36.4)	
6 - 10 years	39 (20.0)	14 (18.2)	25 (21.2)	
11–20 years	47 (24.1)	14 (18.2)	33 (28.0)	
>20 years	35 (18.0)	18 (23.4)	17 (14.4)	
Education^c^				0.78^¤^
None	30 (16.0)	9 (11.8)	21 (18.9)	
Lower primary	41 (21.9)	16 (21.1)	25 (22.5)	
Higher primary	28 (15.0)	12 (15.8)	16 (14.4)	
Attended high school	67 (35.8)	31 (40.8)	36 (43.0)	
Completed high school	16 (8.6)	6 (7.9)	10 (9.0)	
Tertiary education	5 (2.7)	2 (2.6)	3 (2.7)	

^a^1 response missing; ^b^3 responses missing; ^c^11 responses missing

^d^IQR = inter quartile range

^†^Pearson chi-square test, ^¤^Fisher’s exact test, °Wilcoxon rank sum test

Demographic characteristics cross tabulated by mercury use (users versus non-users) are indicated in the last three columns of Table 1. Mercury use for healing purposes was reported by 78 (39 %) THPs. No statistical significance was found between demographic characteristics of the THPs and mercury use (mercury use versus non-use) since all p-values are greater that the predefined significance level of 0.05. Hence, THPs using and not using mercury had similar demographic characteristics.

Of the 181 THPs that responded to providing alternative names for mercury, 170 (94 %) responded that mercury does not have an alternate name other than its local name, *isigidi*. Of the 11 THPs who stated that the product has an alternative name, only six provided an alternative. Three THPs gave the same alternate name *intsimbi (‘metal’)* while the other three THPs provided alternate names of *umviki (‘protector’), umuthi ogxumayo (‘jumping medicine’)* and *imbekisani* (no English translation). Of the 120 THPs responding to reasons for not using mercury, explanations included not knowing how to use it (*n* = 105; 88 %), it being unsafe (*n* = 10), they don’t believe in it (*n* = 3) and that their ancestors say it must not be used (*n* = 2).

Of the 78 mercury administering THPs, 74 (95 %) stated that they were taught to use it by another THP with three THPs revealing that their ancestors taught them how to use it and one THP was self-taught. Six methods of administration were specified, with oral use being the most common followed by sub-cutaneous implantations (85 % and 59 % respectively; Table 2). Table 3 shows the fourteen reasons for mercury administration with childbirth and protection against guns being the most common (90 % and 50 %, respectively). Four respondents (5 %) stated that they administer it for sterility while three respondents (4 %) stated that they use it on pregnant women.

**Table 2:** Mercury administration methods

Administration method	n (%)
Orally	66 (84.6)
Sub-cutaneous implantation	46 (59.0)
Enema	2 (2.6)
Use in bath	2 (2.6)
Inhalation/facial sauna	1 (1.3)
Licking off hand	1 (1.3)

**Table 3:** Reasons for mercury administration

Reason	n (%)
Childbirth	70 (89.7)
Protection from guns	39 (50.0)
Sterility	4 (5.1)
During pregnancy	3 (3.9)
Protection ritual on house	2 (2.6)
Gynaecological complaints	1 (1.3)
Aphrodisiac	1 (1.3)
Sexually transmitted infections	1 (1.3)
Gastrointestinal: aches and cramps	1 (1.3)
Liver	1 (1.3)
Kidney/bladder	1 (1.3)
Nervousness	1 (1.3)
Aches, pains and swelling	1 (1.3)
Love medicine	1 (1.3)

A bivariate analysis of mercury use for childbirth versus demographic variables and methods of administration (Table 4), revealed no significant association between THP gender and mercury administration for child birth (*p* = 0.71). Furthermore, the use of mercury for child birth was consistently similar across all education levels (*p* = 0.93), types of healing practice (*p* = 0.94), location of the THPs (*p* = 0.59), and the two main methods of administration, oral (*p* = 0.10) and sub-cutaneous implantations (*p* = 0.83). However, a significant difference was observed when years of practice was considered with THPs with experience between 5 and 10 years having a lower percentage of mercury use (69 %) as compared to at least 86 % for other experience groups (*p* = 0.01). However, this difference could be due to random fluctuation.

**Table 4:** Bivariate analysis of mercury use for childbirth/gun protection versus demographic variables and methods of administration

	Childbirth		Protection from guns	
Characteristic	n (%)	*p*-value	n (%)	*p*-value
Gender		0.71		0.34^†^
Female	46 (88.5)		24 (46.2)	
Male	24 (92.3)		15 (57.7)	
Education		0.93		0.06
None	9 (100)		3 (33.3)	
Lower primary	14 (87.5)		4 (25.0)	
Higher primary	11 (91.7)		5 (41.7)	
Attended high school	27 (87.1)		21 (67.7)	
Completed high school	6 (100)		4 (66.7)	
Tertiary education	2 (100)		1 (50.0)	
Years of practice				
≤5 years	31 (100)	0.01	19 (61.3)	0.16
6–10 years	10 (71.4)		8 (57.1)	
11–20 years	12 (85.7)		4 (28.6)	
>20 years	16 (88.9)		7 (38.9)	
Type of healing practice				
Diviner (*Sangoma*)	32 (88.9)	0.94	16 (44.4)	0.26
Herbalist (*Inyanga*)	13 (92.9)		4 (28.6)	
Diviner/faith healer	6 (85.7)		4 (57.1)	
Herbalist/diviner	8 (80.0)		5 (50.0)	
Herbalist/faith healer	4 (100)		3 (75.0)	
Faith healer (*Mthandazi*)	1 (100)		1 (100)	
Diviner/THP trainee	2 (100)		2 (100)	
THP Trainee (*Thwasa*)	2 (100)		2 (100)	
Diviner/faith healer/THP trainee	1 (100)		1 (100)	
Herbalist/diviner/faith healer	0 (100)		0 (100)	
Practice area		0.59		0.72
Durban	61 (88.4)		34 (49.2)	
Pietermaritzburg	9 (100)		5 (55.6)	
Oral administration		0.10		0.76
Yes	61 (92.4)		34 (51.5)	
No	9 (75.0)		5 (41.7)	
Cutaneous implantations		0.83		<0.0001
Yes	41 (89.1)		35 (76.1)	
No	29 (90.6)		4 (12.5)	

The variables “Childbirth” and “Protection from guns” were recorded on a binary scale: Yes = 1 and No = 0. Frequencies (percentages) of “Yes” are reported

All *p*-values were obtained using Fisher’s exact test, except where otherwise indicated

^†^Pearson chi-square test

The use of mercury for gun protection was not significantly associated with the variables gender, education, location, types of healing practice, and practice duration of the THPs (*p* > 0.05; Table 4). Mercury use for gun protection was not significantly associated with oral administration (*p* = 0.76), but was strongly associated with sub-cutaneous administration (*p* < 0.0001). Of the 46 THPs reporting sub-cutaneous administration of mercury, 35 (75 %) administer it for gun protection.

There was consensus from all mercury using THPs (*n* = 78) that as far as they were aware, none of their patients had ever had a bad reaction to the mercury (Table 5). The majority (*n* = 76; 97 %) of the THPs stated that mercury is only safe when administered by trained THPs. On further discussing safety of mercury, 10 (14 %) THPs stated that mercury can be used on its own, while 60 (81 %) responded that there is a need to reduce toxicity by mixing/diluting it with other products. Four THPs stated that it could be used both on its own or one could reduce the toxicity (depending on the situation). Mercury usage is believed to be very significant in South African traditional medicine by 51 (67 %) of the THPs whilst other THPs said it was moderately or not at all significant [18 (24 %) and 7 (9 %) respectively; Table 5].

**Table 5:** THP responses to mercury safety and significance in South African traditional medicine

Safety and significance query	n (%)
Have any of your patients had a bad reaction to the mercury?	
No	78 (100)
Mercury use is only safe if used by trained THPs:	
Yes	76 (97.4)
Mercury can be used^a^:	
(a) Safely on its own	10 (13.5)
(b) I need to reduce toxicity	60 (81.1)
(c) Both (a) and (b)	4 (5.4)
Rate the significance of mercury in South African traditional medicine^b^:	
Very significant	51 (67.1)
Moderately significant	18 (23.7)
Not very significant	7 (9.2)

^a^4 responses missing; ^b^2 responses missing

## Discussion

Heavy metals are a regular and deliberate component of traditional remedies globally and consequential poisoning has been documented in both adults and children [10, 16–18]. Hence identification of key exposed populations is imperative in order to estimate the disease burden of harmful metals [19]. Despite global efforts to reduce or eliminate human mercury exposure, mercury in ritualistic practice as a potential source of exposure remains poorly documented [2].

This is the first study to estimate the prevalence of mercury use and describe methods and practice reasons in South African traditional medicine. Mercury in South African traditional medicine is colloquially referred to as *isigidi* which literally means ‘millions’. In our study, 39 % of the THPs interviewed stated that they administer mercury for healing purposes however this finding is cumulative with 95 % of the mercury-using THPs stating that they learnt how to use it from fellow THPs. The potential mercury exposure to THPs is an unregulated and undocumented occupational hazard. The possible mercury spillage during use either at the THP practice or in the home of the end user is cause for concern. In such occurrences, spilled mercury may persist in the flooring for several months, thereby enabling an environment of chronic vapour exposure [20]. Moreover, children may be at higher risk of mercury vapour inhalation because as the vapour settles on the floor, it is in closer proximity to the crawling infant or walking toddler’s respiratory system [21].

The THPs and end users of the mercury are also at risk from mercury exposure via various administration techniques identified in this study. Although during the handling of mercury, skin contact may cause only moderate symptoms such as skin irritation or dermatitis [2, 6], exposure to mercury vapour whilst administering to patients may place THPs and their patients at risk from mercury toxicity.

The most common route of mercury administration that THPs reported was orally. Ingested elemental mercury is poorly absorbed in the gastrointestinal tract (< 0.01 % of the dose). However, a compromised mucosal barrier of the gastrointestinal tract may allow for augmented bioavailability [6]. *Ukugcaba* is an administration technique which typically describes making incisions (sub-cutaneous implantations) and then rubbing traditional medicine into them. The practice of *ukugcaba* for mercury administration was reported by 46 (59 %) of the 78 mercury using THPs. Although poorly absorbed dermally through intact skin, reports of mercury toxicity due to subcutaneous mercury exposure have been documented [22, 23]. Therefore, subsequent to subcutaneous mercury exposure, prevention of systemic absorption is imperative [22]. One THP stated that an administration technique used was inhalation/facial sauna which would put the patient at direct risk of mercury vapour inhalation.

Nearly 90 % of the mercury-using THPs revealed that they administer it for childbirth however the aspect of childbirth (induce labour, pain relief, etc.) was not detailed. Elemental mercury can readily cross the placental barrier thus the developing foetus can be exposed to mercury from the pregnant women's body through the placenta [1, 24]. Furthermore, infants may be exposed to mercury from breast milk [1].

In certain cultural groups, mercury has been documented to allegedly provide personal and/or household protection by warding off evil spirits [4, 11]. Similar results were shown in our study whereby two THPs revealed mercury was used to perform protection rituals on houses. Our study is the first to report on the use of mercury for protection against guns, with the mode of administration for this purpose being mostly by sub-cutaneous implantations. One THP stated (*pers comm*.) that this protection from guns is used by those working within the local minibus taxi industry. This is not surprising as the informal minibus taxi industry, rooted within the informal sector of South Africa, is known for conflict and violence in some areas [25, 26].

The fact that the majority of THPs stated that mercury is only safe to use when administered by THPs and that 81 % responded that there is a need to reduce its toxicity before use implies that there is an awareness of its potential to cause harm. However the reduction of toxicity before use also insinuates that there is a handling of the mercury by the THP or end user before administration which may increase exposure. This study has several limitations. As this study was intended to gather formative data we did not attempt to quantify mercury exposure or administration dosage. Furthermore, the non-random sample may not be representative of THPs in KwaZulu-Natal Province or the rest of the country. Nonetheless, the significance of mercury use in South African traditional medicine reported by THPs in this study implies an established cultural practice. The results of this study were relayed back to the participating THPs, training regarding mercury exposure and harms was provided and safer alternatives were encouraged. Knowledge dissemination with fellow THPs as well as their patients was emphasized.

## Conclusion

This study has identified population groups at risk of mercury exposure. In agreement with the U.S Environmental Protection Agency [4], the promotion of health and well-being while respecting traditions and community autonomy is advocated. Cultural diversity awareness promotion for healthcare professionals is imperative as the knowledge regarding ritualistic mercury use will allow timely and accurate diagnosis of signs and symptoms of mercury poisoning, especially in the light of mercury exposure to pregnant women. Furthermore knowledge regarding traditional medicine mercury exposure and subsequent harm to both mother and foetus should be incorporated into prenatal education sessions.
